# Monitoring Neuronal Network Disturbances of Brain Diseases: A Preclinical MRI Approach in the Rodent Brain

**DOI:** 10.3389/fncel.2021.815552

**Published:** 2022-01-03

**Authors:** Annemie Van der Linden, Mathias Hoehn

**Affiliations:** ^1^Bio-Imaging Lab, Department of Biomedical Sciences, University of Antwerp, Antwerp, Belgium; ^2^μNEURO Research Centre of Excellence, University of Antwerp, Antwerp, Belgium; ^3^Research Center Jülich, Institute 3 for Neuroscience and Medicine, Jülich, Germany

**Keywords:** neuronal networks, structural connectivity, functional connectivity, neurodegenerative diseases, Alzheimer's disease, stroke, Huntington's disease, rodents

## Abstract

Functional and structural neuronal networks, as recorded by resting-state functional MRI and diffusion MRI-based tractography, gain increasing attention as data driven whole brain imaging methods not limited to the foci of the primary pathology or the known key affected regions but permitting to characterize the entire network response of the brain after disease or injury. Their connectome contents thus provide information on distal brain areas, directly or indirectly affected by and interacting with the primary pathological event or affected regions. From such information, a better understanding of the dynamics of disease progression is expected. Furthermore, observation of the brain's spontaneous or treatment-induced improvement will contribute to unravel the underlying mechanisms of plasticity and recovery across the whole-brain networks. In the present review, we discuss the values of functional and structural network information derived from systematic and controlled experimentation using clinically relevant animal models. We focus on rodent models of the cerebral diseases with high impact on social burdens, namely, neurodegeneration, and stroke.

## Introduction

Today, most diagnosis and investigations into the pathophysiology of brain diseases rely heavily on modern imaging techniques. Many different non-invasive imaging modalities provide findings, important to foster understanding of underlying mechanisms, particularly for neurodegenerative diseases and stroke. Thus, positron emission tomography (PET) allows to describe metabolic disturbances and altered receptor density. Magnetic resonance imaging (MRI) characterizes lesion location and tissue alterations like loss of white matter or neurodegeneration. Functional MRI accesses brain activation of tissue areas responding to external stimuli, thus integrating functional deficits and functional restitution into concepts of plasticity potential after disease (Grefkes and Fink, 2014).

Resting-state fMRI (rsfMRI) and tractography by diffusion tensor imaging (DTI) take the understanding to a further new level by disclosing neuronal networks and how they are affected, and by extending the view from the primary lesion focus to the whole brain networks. These imaging techniques permit to elucidate at macroscale the responses of the functional and structural whole-brain networks to the disease, thereby permitting to include observations of distal, whole-brain disturbances as well as of regenerative capacity of the brain. They have by now found wide clinical application for Alzheimer's disease (Acosta-Cabronero et al., 2012; Badhwar et al., 2017), but also more recently for stroke studies (Behrens and Johansen-Berg, 2005; Bonkhoff et al., 2020).

In our present review, we discuss the contribution of resting-state fMRI and DTI tractography to systematic preclinical animal experiments with our focus on the two major fields of brain diseases with high impact on social burdens, namely neurodegenerative diseases and stroke. Application of rsfMRI and DTI tractography are mostly found in rodents of clinically relevant models of neurodegenerative diseases and stroke, thus forming the framework of our present review. All DTI based results, limited to microstructural tissue characterization without explicit tractography analysis are excluded; they do not contribute information on network conditions and are beyond the scope of the present review.

We will first introduce the methodological basis of both imaging techniques and present their value in application to the healthy rodent brain. In the main body of the review, we will discuss the important deficits as well as spontaneous or treatment-induced improvements of the functional and structural neuronal networks during neurodegenerative diseases or stroke. We aim on emphasizing on novel information on the dynamics and underlying mechanisms of the deranged and regenerating brain networks, generated with either structural or functional network investigations.

## How are Networks Determined

The study of dynamic brain changes under normal conditions such as during development or learning or after brain disease and/or recovery requires non-invasive *in vivo* neuroscientific tools in order to allow for comparison of different time points and states of the brain, ideally during longitudinal studies. Thus, different mechanisms like axonal sprouting forming new connections (Granziera et al., 2007) or plasticity-triggered remapping (Silasi and Murphy, 2014) can be unraveled. Changes occurring at the cellular level lead to local circuit changes but also influence whole-brain network systems. Imaging tools with mesoscopic spatial resolution provide important insights into these whole-brain network changes in both time and space.

The following paragraph focuses on magnetic resonance imaging modalities as mesoscopic neuroscience tools and introduces diffusion MR imaging and resting-state fMRI. Diffusion MRI is a suitable tool for the detection of structural connections between different brain regions (structural connectome) while resting-state functional MRI is ideal for the observation of functional connectivity networks across the whole brain (functional connectome).

### Functional Connectivity

Resting-state fMRI (rsfMRI) is based on increase in blood oxygenation level initiated by changes in blood flow and volume in active brain areas. Small fluctuations in this blood oxygenation level give rise to small MRI signal intensity changes in the low frequency range below 0.1 Hz. This BOLD (Blood Oxygenation Level Dependence) MRI signal, first described by Ogawa et al. (1990), led to the first observation by Biswal et al. (1995) of the spontaneous fluctuations across the whole brain. This imaging strategy deciphers signal changes over time. Thus, neuronal nodes are detected in the rsfMRI data sets as regions with synchronous activity during resting brain condition, forming functional networks (“what fires together, wires together,” Hebbian rule). As no external stimuli are applied, rsfMRI is the ideal imaging tool to study spontaneous connectivities and to detect changes thereof under pathological conditions. Such results are achieved with the acquisition of a large series of typically several hundred identical images, recorded rapidly within a defined time window of a few minutes. The temporal signal fluctuations over time are then analyzed on a voxel basis, and the resulting time curves of different voxels are compared for their intrinsic similarity. Similarity is determined by cross correlation analysis. An excellent overview of the methodological requirements is given in detail in Pan et al. (2015).

There are three major analysis strategies for functional brain mapping. The seed-based analysis typically presents the results in so-called z-score matrices with every matrix cell representing the correlation strength between two voxels in the brain. In independent component analysis, clusters of pixels with closely similar high correlation values are mapped together onto anatomical brain background for visualization. Graph theoretical approaches build on the seed-based analysis describing various seed characteristics such as node strength and degree, and focusing on whole-brain characteristics, e.g., density, modularity, cluster coefficient, and hubness (for an excellent review on graph theoretical approaches cf. Hillary and Grafman, 2017).

The rsfMRI data are, due to the hemodynamic character of the BOLD signal, only indirect surrogate markers of neuronal activity. However, in a recent report, Ma et al. (2016) compared rsfMRI data with simultaneously recorded neural activity, measured with optical imaging of calcium-sensitive fluorophore GCaMP. In that study, the authors were able to demonstrate a direct and strong coupling between the rsfMRI hemodynamic pattern and the excitatory neural activity, thus confirming the rsfMRI data interpretation as reliable neuronal network activity.

### Structural Connectivity

The method of choice for the *in vivo* determination of structural connections between individual brain areas is fiber tracking. Fiber tracking uses diffusion tensor imaging (DTI) (Behrens and Johansen-Berg, 2005) or the more advanced diffusion spectrum imaging (DSI), both based on diffusion-weighted MR imaging. This MRI modality exploits the diffusivity of water molecules in aqueous environment and takes advantage of the preferential mobility of water molecules along myelinated axons, compared to the hindered mobility perpendicular to the myelin sheaths. Complex mathematical post-processing of DTI or DSI data results in extraction of preferential fiber directions from one voxel to its neighboring voxels. DSI even distinguishes between crossing or touching fiber bundles in whole-brain networks (Wedeen et al., 2005). Graph theoretical approaches, introduced above for rsfMRI data processing, can also be applied on diffusion-weighted imaging data yielding structural connectome data of the entire brain.

This diffusion-based MR imaging sequence has very high demands on excellent signal-to-noise in small animal studies, as requirement of high spatial resolution results in small voxel sizes. Fiber tracking has successfully been applied particularly to study structural network abnormalities in neurodegenerative disease models and in regeneration studies after experimental stroke in rodents (cf. below; sections Network Changes in Neurodegenerative Diseases and Network Changes after Stroke).

As fiber tracking heavily depends on complex data post-processing of the voxelwise diffusion behavior, it has been of great interest to validate fiber tracks defined by DTI with independent histological analysis. For this purpose, postmortem high resolution diffusion MRI was directly and quantitatively compared with histological measurements of rat brain myeloarchitecture with highly reliable, stable agreement between both techniques (Leergaard et al., 2010).

## Networks in Healthy Brains

### Anesthesia Conditions

*In vivo* MRI studies on small animals requires anesthesia and head fixation to minimize animal distress and prevent image blurring by movements during the scans. While deep anesthesia is acceptable for structural MRI scanning, functional brain studies require light sedation condition in order to minimize the anesthetic effect on the physiological state, in particular the brain activity. Several labs investigated the influence of different anesthesia protocols on functional connectivity results (Kalthoff et al., 2012; Grandjean et al., 2014a; Jonckers et al., 2014). Kalthoff et al. (2012) compared deep isoflurane anesthesia with medetomidine sedation and found that functional connectivity in rats revealed substantially more functional network structures under light sedation. Jonckers et al. reported similar functional connectivity (FC) in awake mice and under isoflurane anesthesia, but much reduced FC strength under urethane and alpha-chloralose anesthesia (Jonckers et al., 2014). In a careful comparison of the effects of isoflurane, propofol, and urethane anesthesia and medetomidine sedation on FC patterns in the mouse brain, Grandjean et al. (2014a) concluded that a mixture of low dose isoflurane and medetomidine best “retained strong correlations both within cortical and subcortical structures… ..rendering this regimen an attractive anesthesia for rsfMRI in mice.” The wide majority of later studies have followed these authors' anesthesia protocol of low dose isoflurane with medetomidine or slight variations thereof (Shah et al., 2016b; Green et al., 2018; Egimendia et al., 2019), as convincingly demonstrated in a recent multi-center study on mouse rsfMRI (Grandjean et al., 2020). However, understanding the differential effects of anesthetics on brain networks and their interaction is essential when interpreting fMRI data recorded under specific physiological and pathological conditions (Bukhari et al., 2017).

Recent efforts have been made to develop rsfMRI protocols for awake rodent studies (Bergmann et al., 2016; Ma et al., 2018; Paasonen et al., 2018; Tsurugizawa et al., 2020). However, these protocols often need special animal cradles (Madularu et al., 2017) and require extensive acclimation periods of more than a week, also sometimes including preparatory surgery for implantation of head posts for fixation. Despite the attention to careful acclimation training periods, data on animal stress levels is not always reported. Future better understanding about the robustness of such protocols is expected from a recently established open database of rsfMRI in awake rats (Liu et al., 2020).

### Mice Strain Differences

Mouse models of a specific neurological disease are often generated using different background strains, which raises the question whether the observed effects are specific to pathology or depend on the used strain. Shah et al. (2016b) used a combination of *in vivo* rsfMRI and PET on three different mouse strains (C57BL/6, BALB/C and SJL) to evaluate differences in FC and brain glucose metabolism, as measures of neuronal activity. Comparing FC and [18F]-FDG data showed that strain-dependent differences in brain activity exist for several brain networks where FC was highest in the SJL model, followed by the BALB/C, and finally the C57BL/6 mice had the lowest FC. These results demonstrate that functional processes might be different between mouse strains, which may reflect the known behavioral differences between strains. Another possibility is that the differences between the strains is due to the strain dependent differential effects of medetomidine, the anesthesia used in this study.

Wang et al. (2020) acquired DTI based connectomes and microscopic MRI atlases for diverse mouse strains and both sexes, unraveling not only variability but also heritability in their connectome. They generated whole-brain structural MRI and diffusion-based structural connectomes for four diverse isogenic lines of mice (C57BL/6J, DBA/2J, CAST/EiJ, and BTBR) at a spatial resolution 20,000 times higher than human connectomes. Nearly 150 of the connection profiles were statistically different between the C57BL/6J, DBA/2J, and CAST/EiJ lines. Recently, Karatas et al. (2021) investigated strain specific patterns of brain structural and functional connectivity in *C57BL/6N* and *BALB/cJ* mouse strains. They demonstrated that structural dissimilarities were accompanied by specific FC patterns, emphasizing strain differences in frontal and basal forebrain functional networks as well as hubness characteristics. RsfMRI data further indicated differences of reward-aversion circuitry and default mode network (DMN) patterns. These studies show on the one hand an important confounding factor to be considered while constructing preclinical disease models, as they would also reflect the inherent strain characteristics and strain-specific vulnerability of brain circuitry to pathological mechanisms. On the other hand, they demonstrate that these MRI methods are highly sensitive to detect differences in genetic background and relevant behavioral phenotype. In application to genetic manipulations, these functional imaging modalities may contribute to a better understanding of the causal relationship between genetic variation, functional connectome, and behavior.

### Functional Networks

**Figure 1:**
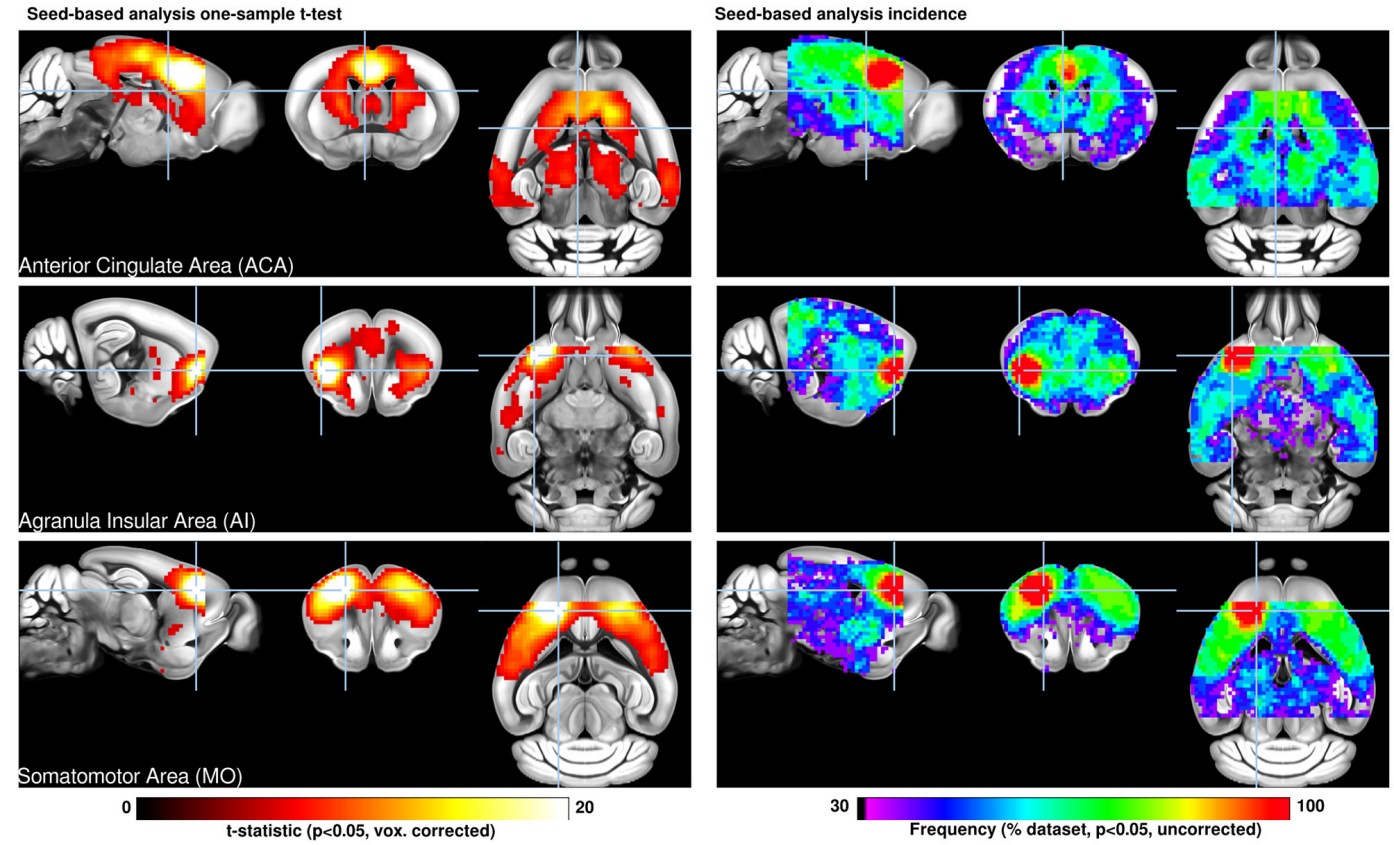
Seed-based analysis (SBA) for three selected seeds positioned in the left hemisphere. One-sample *t*-test maps of individual functional connectivity maps reveal the full extent of SBA-derived resting-state networks in the mouse brain across 98 independent scans. Functional connectivity (FC) related to a seed located in the anterior cingulate area reveals the extent of the mouse default mode network including dorsal caudate-putamen, dorsal thalamus, and peri-hippocampal areas. The seed in the insular area reveals significant FC in dorsal cingulate and amygdalar areas, corresponding to areas associated with the human salience network. Interhemispheric homotopic FC is found relative to the somatomotor seed, together with lateral striatal FC. Overlap maps indicate the percentage of data sets presenting significant FC which recapitulate the features above. Reproduced with permission from Grandjean et al. (2020).

Since the first publication on mouse resting-state fMRI (Jonckers et al., 2011), application of rsfMRI to probe the whole-brain networks in rodents has been rapidly increasing, often times with the motivation to use healthy brain conditions as baseline to better understand disease-triggered alterations. Although the focus of the many individual studies was on varying parts of the brain, Grandjean et al. (2020) recently brought many of these original datasets together to perform an excellent multi-center study, including 17 datasets from different laboratories, earlier already published as separate datasets. Homogenized data analysis was based on the two most widely used brain mapping approaches, i.e., seed-based analysis and independent component analysis. This multi-center study thus provides a rather robust picture of the healthy mouse brain functional networks, based on a very large sample size, not easily achieved under normal conditions in individual labs. The major robust patterns found in almost all datasets include a bilateral, homotopic extension of networks. Several individual networks, often also reflecting anatomical features of human, primate or rat networks have been identified. Here the most prominent networks are the sensorimotor network, the salient network, and the default mode network (DMN; including the anterior cingulate area, the pre-frontal cortex, retrospinal area, dorsal striatum and thalamus, and peri-hippocampal areas) and the latero-cortical network (LCN) antagonistic to midline DMN regions, a homolog to the task positive network (TPN) in humans (Grandjean et al., 2020) (Figure 1).

As behavior, pharmacological manipulations, brain stimulation (deep brain stimulation, optogenetics, DREADDs), and neuropathology can all modulate the rsfMRI results in rodents, this readout represents an important asset in translational preclinical neurosciences. Besides the impact of anesthesia on the FC, the outcome of pharmacological manipulations affecting the FC may be considered as a readout for evaluating the efficacy of drug treatment. Furthermore, using drugs that target specific receptors could help to delineate the neural correlate of FC in relation to the underlying receptor system, pathway, or functional deficit (for an extensive review, see Chuang and Nasrallah, 2017). Shah et al. (2015) performed rsfMRI before and after injection of the cholinergic antagonist scopolamine and observed a dose dependent decrease of FC between brain regions involved in memory. Scopolamine-induced FC deficits were almost completely recovered by the cholinergic agonist Milameline, consistent with the behavioral outcome of the animals, thus providing insight in the neural substrate of the investigated behavior patterns (Shah et al., 2015). This shows that functional connectivity can serve as a readout for a temporary (modified) synaptic activity. In a follow-up study, Shah et al. (2016a) adopted pharmacological modulations that target specific neurotransmitter systems such as the cholinergic and serotonergic system by using their appropriate antagonists. Both impaired the FC of the mouse DMN network similarly, except that cholinergic modulation additionally affected the retrosplenial cortex, demonstrating that both neurotransmitter systems are involved in maintaining integrity of FC within the DMN network in mice. Such pharmacological rsfMRI experiments in animal models can provide insight into the role of specific neurotransmitter systems on functional networks in healthy situations and in neurological disorders.

A recent report by Aswendt et al. (2021a) emphasized the role of the gut-brain axis on the condition of healthy mice. These authors compared the functional networks of mice held under normal SPF conditions with animals raised and held under germ-free conditions. The germ-free animals presented with a profound hyperconnectivity of an approximately doubled functional connectivity strength. Further graph theoretical analysis of these datasets revealed significantly higher values in germ-free animals indicating denser and stronger global network but with less structural organization. These new results demonstrate the substantial impact of bacterial colonization on brain wide function of disease-free naïve brains.

Several investigations on development of brain diseases performed longitudinal rsfMRI studies of their disease models and included a healthy control group of mice over the same longitudinal observation period up to several months. Thus, Shah et al. (2016c) investigated functional connectivity changes during amyloid plaque deposition in transgenic and wild type (WT) mice during an age span of 3–18 months. Similarly, Grandjean et al. (2014b) reported FC alterations in transgenic mice of cerebral amyloidosis, following animals between 1 and 21 months of age. Although both studies focused on the difference between WT and transgenic animals over time, their reports show a clear, even though small, change of FC strength with age. In a detailed study of FC changes during a life span, Egimendia et al. (2019) were able to show a clear inverse U-shape curve of the FC strength during the period of 2–13 months of age. Although the effect was hardly visible for whole-brain average analysis, and the changes were of variable intensity expression for individual anatomic seeds, a clear pronounced inverse U-shape was noted for separate networks such as cortical, subcortical, and the default mode network. In all cases, the inverse U-shape curve presented its maximum at 8–9 months of age, followed by continuous decrease during later aging phases. This age dependence has important consequences for longitudinal disease development studies, as some functional network strength alterations (described typically as hyper- or hypo-connectivity relative to pre-disease state) may actually be due to age-dependent variations already.

### Dynamic Functional Connectivity Patterns: Functional Brain States

Most functional connectivity studies have assumed stationary interactions between brain regions, not considering dynamic aspects of network organization. The first observations of non-stationary FC in rodents was reported by Majeed et al. (2011) who found reproducible, quasi-periodic spatiotemporal patterns of BOLD fluctuations in the anesthetized rat. These patterns were also observed in humans (Majeed et al., 2011), macaques (Hutchison et al., 2013), and were finally confirmed in mice (Belloy et al., 2018) revealing similar dynamic pattern changes across species. The most common spatiotemporal patterns consisted of an activation alteration between areas comprising the DMN and the TPN. This novel approach for probing the spontaneous dynamic network activity of the brain has implications for the interpretation of conventional studies and may increase the amount of information that can be obtained from neuroimaging data. Grandjean et al. (2017a) evaluated the feasibility and research potential of mouse dynamic FC using the interventions of social stress or anesthesia duration as two case-study examples. By combining a sliding-window correlation approach, several dynamic functional states (dFS) with a complex organization were identified, exhibiting highly dynamic inter- and intra-modular interactions. Each dFS displayed a high degree of reproducibility upon changes in analytical parameters and across datasets. They fluctuated at different degrees as a function of anesthetic depth and were sensitive indicators of pathology as shown for the chronic psychosocial stress mouse model of depression. Multimodal studies in animals have provided key information about the neural basis of these network dynamics. Using simultaneous rsfMRI and microelectrode recording in rats, Thompson et al. (2013) showed that BOLD sliding window connectivity at least partially reflects underlying LFP coordination, particularly in the theta, beta and gamma frequency bands. It was also shown that anesthesia has a differential effect on static and dynamic functional connectivity (Tsurugizawa and Yoshimaru, 2021). It is expected that dynamic functional states will make a major contribution to future information integration and processing in the healthy and diseased brain. Although there is so far no rodent stroke study involving dynamic functional network changes, a recent clinical publication demonstrated the added value of exploring temporal variability of functional connectivity. Hu et al. (2018) claimed that inclusion of temporal variability of functional brain networks may even help predict motor recovery after stroke.

### Structural Networks

Structural network information obtained with fiber tracking by diffusion-weighted MRI had quickly become of great interest after the first reports of diffusion MRI's sensitivity to white matter orientation in the spinal cord (Moseley et al., 1991). To determine the reliability of DTI fiber tracking detection of smaller structures, several authors recorded *ex vivo* fiber tracking data of rat (Leergaard et al., 2010; Gyengesi et al., 2014) and mouse brain (Jiang and Johnson, 2010; Calamante et al., 2012; Richards et al., 2014; Calabrese et al., 2015) at micrometer resolution. These results were, however, achieved at the cost of scan times of several hours, in an extreme case up to 235 h (Calabrese et al., 2015). In all these reports, DTI based fiber tracking results were compared with various histological staining techniques for white matter or with virus-mediated fluorescent protein expression (Richards et al., 2014) or neuronal tracers from the Allen Brain Atlas (Calabrese et al., 2015). These detailed validation studies concluded reliable detection of white matter fiber connections by DTI fiber tracking.

**Figure 2:**
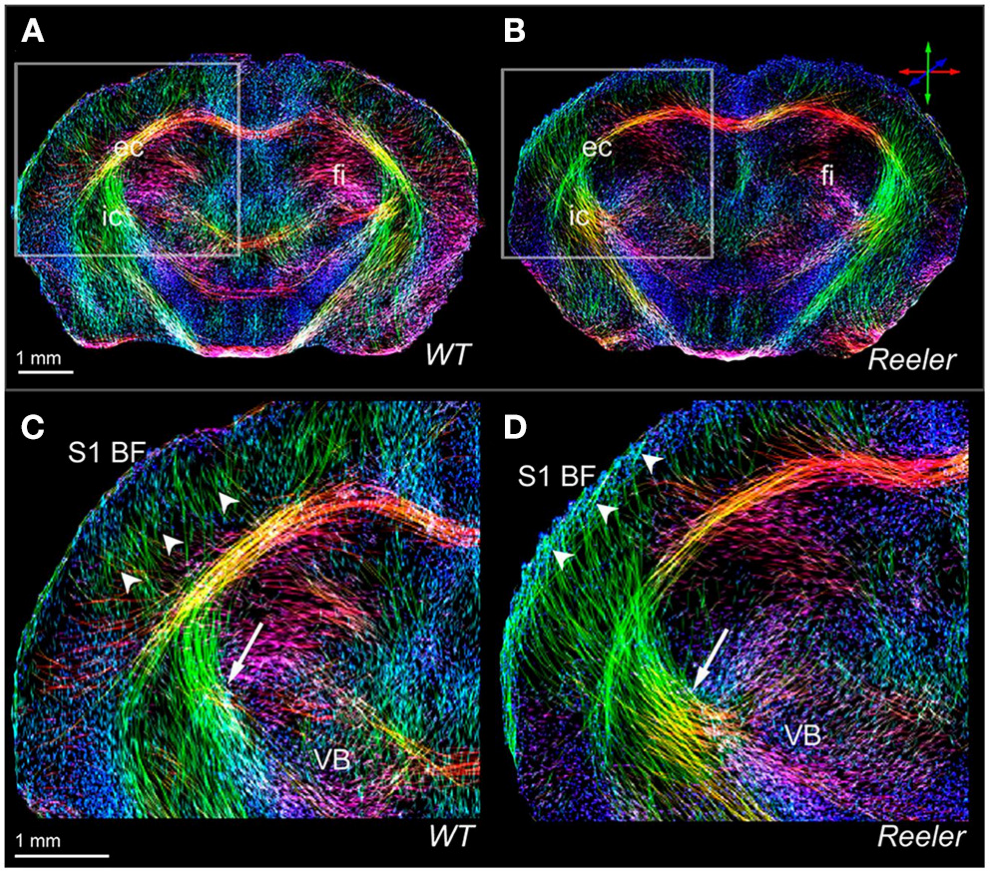
High-resolution views of the thalamocortical fiber architecture of the wild-type and *reeler* mouse brain. *In vivo* high-resolution fiber maps of normal **(A,C)** and *reeler* **(B,D)** brain captures major features of normal **(C)** vs. remodeled **(D)** subcortical (arrows) and thalamocortical (arrow heads) fiber trajectories of the somatosensory thalamocortical pathway. The magnified views **(C,D)** illustrate the fiber bundles emerging from the ventrobasal thalamic nucleus (VB) to target S1BF (S1 barrel field); they cross the internal capsule (**C,D**, arrow) and gather beneath the cortical gray matter before extending intracortically up to presumptive layer IV of the wild-type S1BF (**C**, arrow heads). Note that the *reeler* thalamic fibers **(D)** are defasciculated to form a broader array projecting diagonally (**D**, arrow) into the cortical areas. Reproduced with permission from Harsan et al. (2013).

At much shorter scan times, compatible with true *in vivo* conditions but still at excellent spatial resolution, various connection pathways in the mouse brain were analyzed with DTI fiber tracking. Harsan et al. (2010) reported the *in vivo* resolution of the connection network of various limbic areas, and connections of the amygdala with nucleus accumbens and with visual and auditory cortex. In a comparison of naïve wild type and reelin mice, Harsan et al. (2013) demonstrated at highly impressive resolution the thalamocortical pathway (Figure 2). Their fiber tracking results further allowed to unravel the laminar distortions in the reelin mouse, leading to remodeling of the thalamocortical projections and thus revealing extensive brain plasticity. Diffusion spectrum imaging (DSI), permitting a more complex analysis of fiber circuits for a reliable discrimination of crossing fibers in the brain was first applied in 2012 to rat brain structural analysis. At excellent resolution, Kim et al. (2012) presented details of the thalamocortical pathway and cortico-cortical connections in the naïve rat brain. These authors, furthermore, used forepaw stimulated fMRI to detect the cortical primary sensory representation area for determination of the corresponding thalamocortical pathway. Using alternatively the anatomical presentation of the whole primary sensory cortex lead to additional fiber connections, not included in the specific stimulus-based S1 presentation. In a follow-up investigation, Po et al. (2012) reported reorganization in the internal capsule after stroke, followed by transhemispheric new fiber connections in parallel with functional recovery of the sensorimotor cortex after forepaw stimulation.

Corresponding to the sensitivity of functional connectivity to aging, also structural changes during development and normal aging processes may have to be considered in longitudinal studies or comparison of groups at differing age, but such tractography studies are still missing in the literature. However, Mengler et al. (2014) showed that in rats myelination continues to increase up to 6 months of age in the cortex, and up to 3 months of age in the striatum. In a follow-up study on mice, Hammelrath et al. (2016) demonstrated again a continuing myelination increase in cortical layers during the first 6 months of lifetime. Interestingly, the cortex thickness decreased during this period of brain volume increase, resulting in rather constant cortex volume.

## Network Changes in Neurodegenerative Diseases

Alzheimer's disease (AD) is a progressive age-related neurodegenerative disease, which has become the most common form of dementia in elderly populations. The diagnosis of this pathology is confirmed only several years after the beginning of the disease and no treatments exist until now. In order to find effective treatments, the disease evolution must be understood from the very early stages, preferentially before accumulation of plaques and before irreversible damage has occurred. To date, MRI is the only methodology able to provide a non-invasive insight into the large scale functional and structural network architecture providing the unique possibility to study neurodegenerative diseases as connectivity diseases at a connectome level.

To broaden and complement the studies on AD we will include studies on Huntington's disease (HD), another proteinopathy with a different pathological protein deposition (mutant huntingtine), and different affected brain regions (striatum and cortex). Similar studies on Parkinson's disease (PD) models are almost absent.

### Network Changes in Alzheimer's Disease

In general, current transgenic (tg) AD mouse models are either amyloidosis models, recapitulating only the amyloid β (Aβ) plaque formation from the disease, or tau models, recapitulating only the tau phosphorylation and tangle formation, or multiple tg models combining both pathologies. Most single tg models do not recapitulate the massive neuronal loss and the consequent substantial enlargement of the ventricles as seen in patients, but they do display cognitive deficits, even early in the pathology. The 5X tg AD models on the other hand display severe neuronal loss. A complete overview and full AD related characterization of tg AD models an be found on ALZFORUM.org.

As brain-wide neural networks and white matter are affected in AD, rsfMRI and DTI techniques were already included in patient studies (Reijmer et al., 2012; Badhwar et al., 2017) and in preclinical MRI studies of tg AD animal models. Obviously, preclinical studies on animal models are best suited for detection of early biomarkers and markers for early disease progression. They also permit subsequent cellular and molecular investigations to further investigate the pathological underpinning of aberrant rsfMRI and DTI outcomes.

### Investigation of Amyloidosis Models

**Figure 3:**
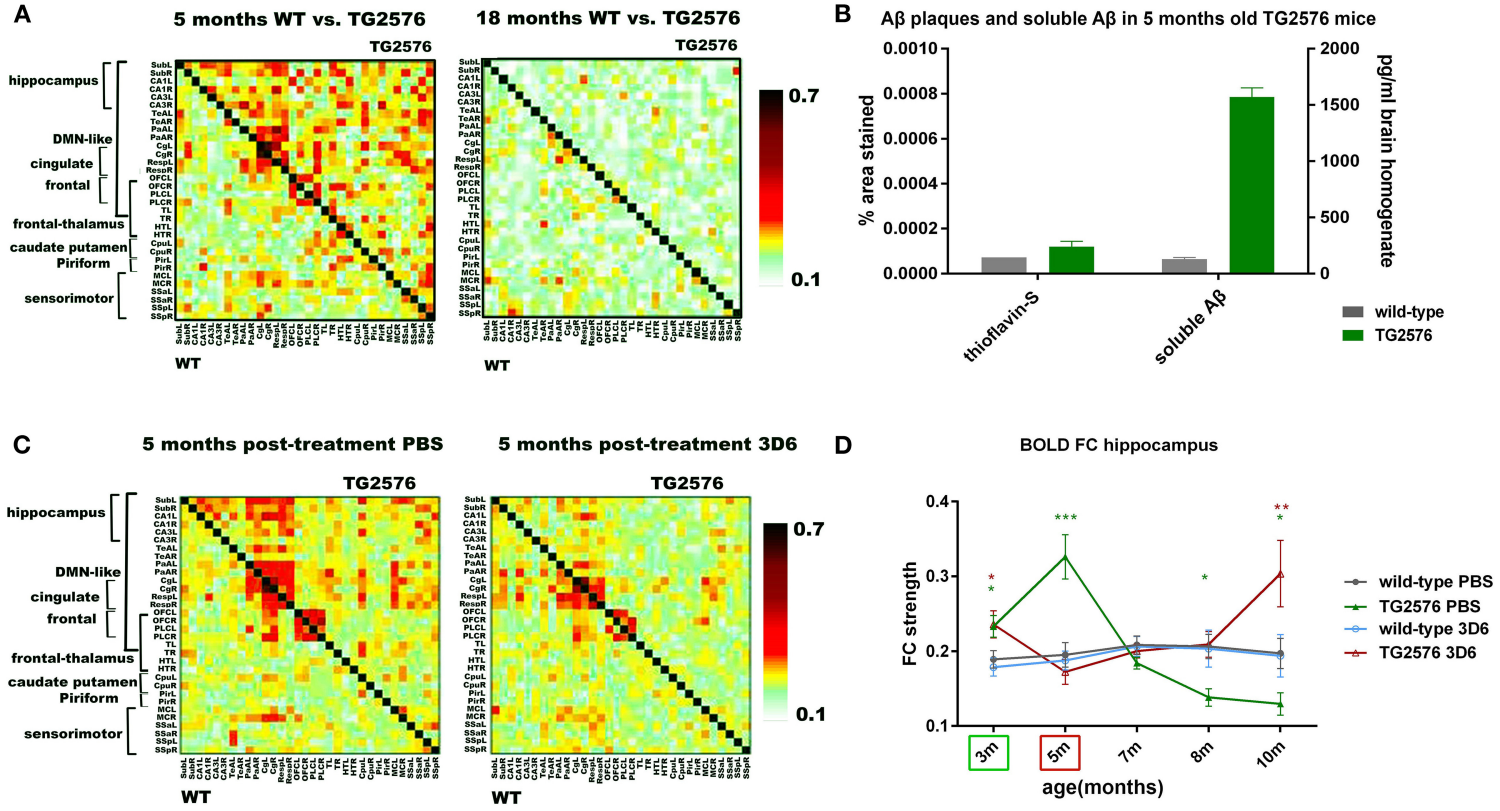
Anti-amyloid beta (Aβ) antibody treatment prevents early hyperconnectivity. **(A)** Mean BOLD FC matrices show z-transformed Pearson correlations between pairs of brain regions of wild-type (WT) (lower half of matrix) and TG2576 tg mice (upper half of matrix) at 5 and 18 months of age. At 5 months of age, TG2576 mice show hyperconnectivity in most brain regions, which evolves to decreased BOLD FC at 18 months of age. **(B)** 5 months old TG2576 mice show no Aβ plaques, as shown on the left Y-axis of the graph (% area stained by Thioflavin-S ± standard error). Soluble Aβ 42 is increased at 5 months of age, as shown on the right Y-axis (pg/ml Aβ42 concentration ± standard error, measured by ELISA). **(C)** Chronic treatment with the anti-Aβ antibody 3D6 prevents BOLD hyperconnectivity in TG2576 mice. Mean BOLD FC matrices show z-transformed Pearson correlations between pairs of brain regions for saline-treated and 3D6 treated mice. **(D)** Graph shows evolution of hippocampal BOLD FC for saline-treated and 3D6 treated wild-type and TG2576 mice (FC ± standard error). Treatment started at 3 months (indicated by the green box) until 5 months (indicated by the red box). Five months after ending the treatment, BOLD hyperconnectivity in the hippocampus reoccurred in 3D6 treated mice, strongly suggesting a role of soluble Aβ in hyperconnectivity. Adopted from Shah et al. (2016c). *P value < 0.05; **P value < 0.01; ***P value < 0.001.

Amyloidosis models recapitulate the Aβ plaque formation in AD which is occurring in patients long before the disease diagnosis and could therefore be considered as early AD models. However, the non-linear correlation between Aβ plaque deposition and dementia has led to a shift of the amyloid cascade hypothesis toward the soluble form of Aβ, which exerts synaptotoxic and neurotoxic effects (Mucke and Selkoe, 2012). Several tg mouse models of amyloidosis show cognitive dysfunctions and impaired synaptic functioning before the manifestation of Aβ plaques and thereby support the destructive role of soluble Aβ on neurologic processes that underlie learning and memory. Grandjean et al. (2014b) investigated whether Aβ plaque deposition is preceded by a decline in FC or is a consequence thereof. These authors performed rsfMRI and DTI on the ArcAβ transgenic amyloidosis model and wild-type mice across their life span, starting as early as 1 and 2 months of age while these mice only develop plaques only at 9 m of age. Transgenic mice showed compromised development of FC during the first months of postnatal life compared with WT animals, resulting in functional impairments that affected in particular the sensory-motor cortex already in the pre-plaque stage. These functional alterations were accompanied by microstructural tissue changes, reflected by reduced fractional anisotropy (FA) values, derived from DTI data. This suggests that cerebral amyloidosis in mice is preceded by impairment of neuronal networks and white matter structures. Shah et al. (2016c) performed longitudinal rsfMRI starting at 3 months of age in the tg2576 amyloidosis model which displays abundant plaque formation at 12 months. She discovered early (3 and 5 months) hyperconnectivity of networks (hippocampal and DMN) before Aβ plaques were formed but upon detection of substantially increased soluble Aβ levels in the brain (Figure 3). The toxicity of soluble Aβ destroyed the GABAergic synapses leading to increased excitation according to Busche et al. (2015). Treatment with the mouse Aβ antibody Benzeneuzimab (known to lower soluble Aβ levels), starting before the onset of hyperconnectivity and up to 7 weeks, could postpone this as long as the treatment lasted. Such hyperconnectivity in absence of plaques but upon increased soluble Aβ in the brain was confirmed in the same paper in the PDAPP mouse model of amyloidosis, and later confirmed in three other models as we will show next. In APP^NL−F/NL−F^mice which allow assessing the effects of pathological Aβ without interference of APP-artifacts, Shah et al. (2018) demonstrated cognitive defects at 3-month of age which coincided with hyperconnectivity of the hippocampal, cingulate, caudate-putamen, and default mode networks, altered by early Aβ pathology. Latif-Hernandez et al. (2019) examined the occurrence of behavioral, cognitive and neuroimaging changes in APP^NL−G−F^ knock-in mice (carrying Swedish, Iberian, and Arctic APP mutations) and compared them to APP^NL^ mice (APP Swedish) to dissociate the effects of aggressive Aβ pathology. The behavior at early ages was largely unaffected while rsfMRI revealed increased pre-frontal-hippocampal network connectivity in 3-month-old APP^NL−G−F^ mice, well before prominent amyloid plaque deposition. The above findings demonstrate that soluble Aβ is involved in pathological hyperconnectivity of brain resting-state networks in different transgenic developmental-onset mouse models of amyloidosis. However, the impact of protein overexpression during brain postnatal development may cause additional phenotypes unrelated to AD. To address this concern, Ben-Nejma et al. (2019) investigated soluble Aβ effects on functional resting-state networks in transgenic mature-onset amyloidosis Tet-Off APP tg mice. Tg mice and control littermates were raised on doxycycline (DOX) diet from 3 d up to 3 m of age to suppress transgenic Aβ production. RsfMRI was performed from week 0 (3 m old mice) up to 28 w post DOX treatment. FC analysis demonstrated early abnormal hyperconnectivity in the tg mice compared to the controls at 8 w post DOX treatment, particularly across regions of the default mode network. At this timepoint a 20-fold increase in total soluble Aβ levels was measured. This study confirms the effect of soluble Aβ at adult onset. At week 28 post DOX treatment, tg mice showed an overall hypoconnectivity, coinciding with a widespread deposition of Aβ plaques in the brain. Also, the other studies reported above confirmed that rsfMRI hyperconnectivity was followed by hypoconnectivity once plaque deposition was prominent (Shah et al., 2013, 2016c, 2018; Latif-Hernandez et al., 2019). Moreover, it is important to mention that increased FC was also detected in children carrying the presenilin1 (PSEN1) mutation compared to non-carriers (Quiroz et al., 2015). This confirms the translational value of this preclinical early-stage network hyperconnectivity and even opens the way to potential preventive treatment in genetically disposed AD patients. Kara et al. (2021) performed a longitudinal rsfMRI and DTI study in female tg2576 mice that underwent pre-menopausal bilateral ovariectomy and thus added an additional AD risk factor. They observed a decrease in white matter integrity, and a decrease in rsfMRI FC in ovariectomized vs. sham-operated tg mice. They also observed an increase in FC in ovariectomized compared to sham-operated wild-type mice. Taken together, their results indicated that both, genotype and ovariectomy altered DTI values and rsfMRI outcomes associated with AD.

Besides Aβ plaques, genetic risk factors can influence the functional network outcome. The cholesterol-transporter apolipoprotein ε (APOE) genotype is associated with the risk of developing neurodegenerative diseases. Investigation of the FC in apoE-ε4 carriers showed a marked differentiation in several functional networks at different ages compared with carriers of other apoE isoforms. The causes of such hampered FC are not understood while vascular function and synaptic repair processes are both impaired in carriers of APOE ε4. Zerbi et al. (2014) investigated FC changes in relation with perfusion and synaptic density in apoE4 and apoE-knock-out (KO) mice at 12 and 18 months of age. Compared with WT mice, apoE4 and apoE-KO mice, both showed FC deficits at 12 months, whereas perfusion deficits and reduced postsynaptic density levels were postponed till 18 months of age only in apoE4. This provides evidence for a relation between apoE and brain connectivity, possibly mediated by vascular risk factors and by the efficiency of APOE as synaptic modulator in the brain.

### Investigation of Tau Models

**Figure 4:**
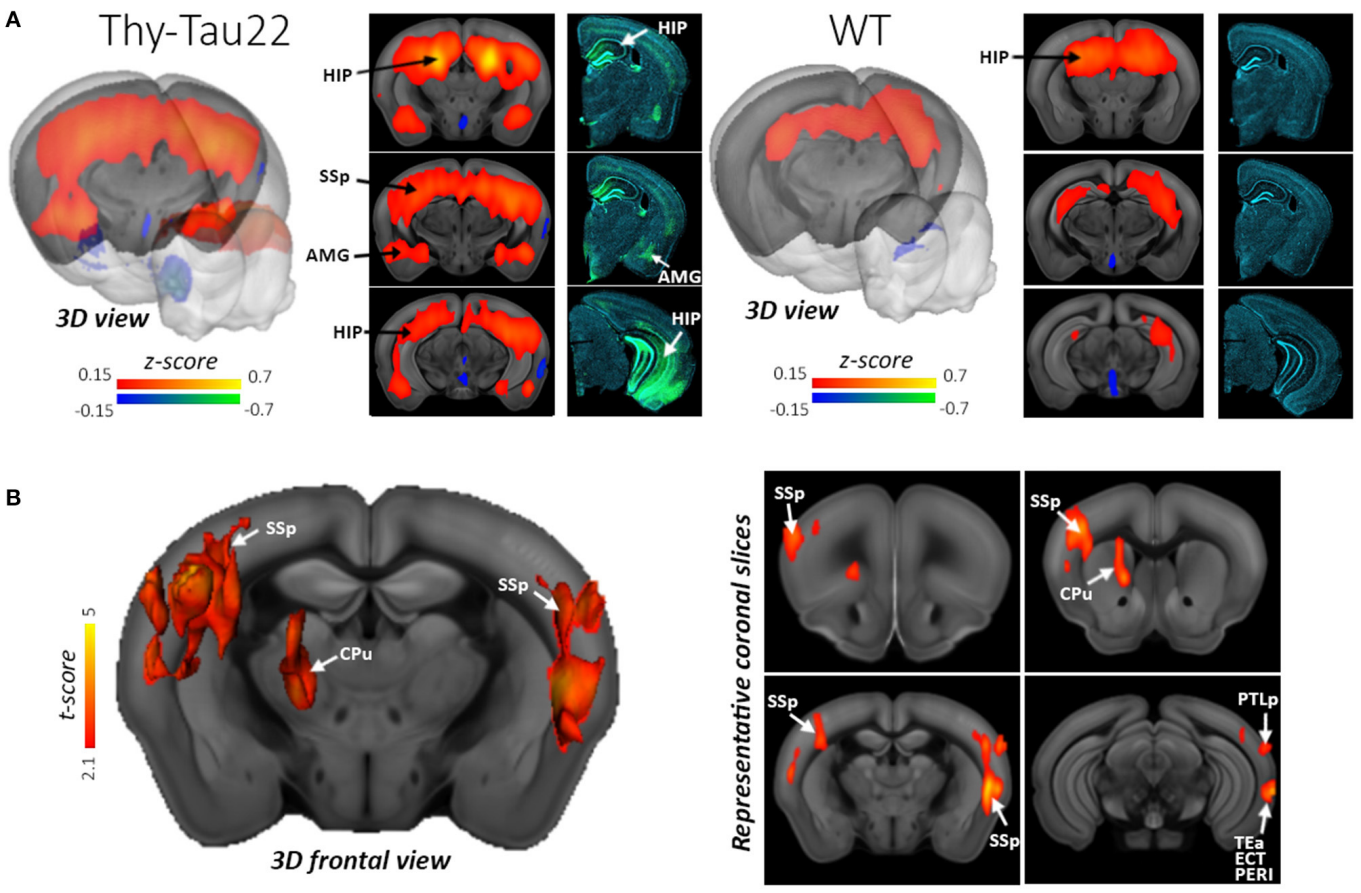
Hyperconnectivity of the dorsal Hippocampus (dHIP) in Thy-Tau22 mice. **(A)** 3D rendering and coronal slices of the seed-based analysis of the dHIP network showing the mean correlation between the mean BOLD signal in the dHIP and all other voxels of the brain, in Thy-Tau22 and wild-type (WT) mice. The color scale represents the strength of the functional correlation normalized with a Fisher z-transformation. Corresponding coronal slices of immunofluorescence staining are shown in the right column in Thy-Tau22 and WT mice: staining of phosphorylated tau (AT8 antibody in green) and DAPI (blue). **(B)** 3D slice representation and maps (right side) of statistically significant differences between the groups (two sample *t*-test, P5 0.01, FDR cluster corrected) showing specific hyperconnectivity of the dHIP in Thy-Tau22 with primary somatosensory cortex (SSp) temporal associative (TEa), posterior parietal associative areas (PTLp), perirhinal cortex (PERI), ectorhinal cortices (ECT) and caudate putamen (CPu). Reproduced with permission from Degiorgis et al. (2020).

Tau models recapitulate another distinct hallmark of Alzheimer's disease, tauopathy, which has been the research focus of clinical trials and preclinical studies. Degiorgis et al. (2020) reported a study on young Thy-Tau22 transgenic mice expressing tauopathy, using rsfMRI and DTI to identify early connectome signatures of the pathology, and related them with histological and behavioral investigations. Before the emergence of cognitive impairments, Thy-Tau22 mice displayed a dysregulation of neuronal networks in key memory regions. The data also demonstrate that connections of hippocampal subregions (dorsal and ventral part) to the amygdala are differently affected. The dorsal hippocampal network showed a strong increase in FC both within the hippocampus and toward isocortical regions, amygdala and caudate putamen in the Thy-Tau22 mice. This hyperconnectivity was consistent with high pathological tau deposition in both hippocampus and amygdala (Figure 4). The authors suggested the coexistence of two intermingled mechanisms of response at the functional connectome level in the early phases of tau pathology: a maladaptive and a likely compensatory response. Detrez et al. (2020) investigated the impact of seeded tau pathology on functional brain connectivity using a fibril-seeded hTau-P301L mouse model. Despite a progressive aggravation of tau pathology across the brain, the major functional networks appeared unaffected up to 15 weeks after seeding and no behavioral changes were detected between seeded and control hTau-P301L mice. Recent reports pointed toward a toxic effect of soluble or oligomeric tau in the spreading of tau pathology in AD. To investigate this further, Green et al. (2019b) studied the effects of expressing human tau repeat domain (tauRD) with pro- or anti-aggregant mutations in regulatable tg mouse models of AD. FC was strongly reduced in transgenic animals in response to the expression of extra TauRD, independent of its aggregation and cognitive decline. This observation points to a novel property of soluble tau, while previous results pointed to a role of aggregation propensity in reaching a pathological state. In contrast to the results on FC, the structural connectivity (based on DTI tractography) was only minimally affected before and throughout treatment. The FC results obtained from the different tau models present further evidence for early tauopathy biomarkers as a potential early-stage drug target by functional networks analysis.

### Investigation Focusing on Triple Transgenic Animal Models

Triple transgenic animal models display temporal- and regional-specific profiles of amyloidosis and tauopathy and could help in disentangling the mutual interactions of the different pathological features and in unraveling their respective MRI readouts. Manno et al. (2019) performed DTI and rsfMRI in 3xTg-AD mice already at 2 months of age, prior to development of intraneuronal plaque accumulation, and showed decreased interhemispheric hippocampal FC while FC in the caudate putamen (CPu) was similar to controls. Liu et al. (2018a) using the same model determined regional homogeneity from rsfMRI data, a measure of local synchronous activity, to discriminate the effects of Aβ and tau on neuronal network activity throughout the brain. Compared to age-matched WT mice, 6- to 8-month-old 3 × TgAD mice exhibited increased regional homogeneity in the hippocampus and parietal and temporal cortices, regions mostly associated with tau in the absence of apparent Aβ pathology in these regions. By 18–24 months of age, 3 × TgAD mice exhibited extensive tau and Aβ pathologies involving the hippocampus and multiple functionally related brain regions, with a spatial expansion of increased local synchronous activity to include those regions. These findings show that age-related brain regional hypersynchronous activity is associated with early tau pathology in this mouse model. This is consistent with a role for early tau pathology in the neuronal circuit hyperexcitability responsible for increased risk for epileptic seizures and contributing to neuronal degeneration in AD. In a preliminary study on a small number of the five-familial AD (5XFAD) tg mice, which exhibits accelerated pathology and neuronal loss, Kesler et al. (2018) determined rsfMRI and DTI connectome properties and measured their topological properties by applying graph theoretical analysis. As the connectomes show some correspondence with results observed in patients with AD, future studies might help to better understand the molecular mechanisms underlying connectome disruption in AD.

### Investigation of TgF344-AD Rat Model of AD

The TgF344-AD rat model of AD has been described to manifest the full spectrum of AD pathology similar to human AD, i.e., progressive cerebral amyloidosis (6 months), tauopathy with tangles, neuronal loss (15 months) and age-dependent cognitive decline (starting at 6 months). AD-related pathology in female TgF344-AD rats was examined longitudinally between 6 and 18 months by rsfMRI to evaluate FC and by DTI to assess the microstructural integrity (Anckaerts et al., 2019). Decreased FC was the earliest detectable hallmark in this model and preceded significant Aβ plaque deposition and the presence of (DTI) structural tissue changes in the affected regions. The progressive aggravation of neuroimaging abnormalities, as well as the regions affected in the TgF344-AD rat model are in line with previous human AD research, which show pathological changes in posterior, hippocampal areas and temporal lobes. Tudela et al. (2019) performed rsfMRI in male TgF344-AD and control rats every 3 months, starting at 5 months of age and continuing to 18 months of age. The rats received repeated training sessions before the first MRI time point, to enable testing of their cognitive performance with a task at each subsequent time point. Using high dimensional ICA they focused on FC between subcomponents of the DMN and showed a lower FC within and between the investigated subnetworks in the TgF344-AD rat starting at 5 months, and different temporal evolution in a specific DMN subnetwork consistent with AD patient findings. The study also shows that the training and the repeated tasks before each MRI are a cognitive intervention that modifies the evolution of functional networks and their connections differently in WT and in TgF344-AD rats. The same rsfMRI dataset with the addition of diffusion-weighted MRI was processed by the same group with a graph theory approach, less used in animal models but often used in human studies and thus with high translational relevance. In this way structural and functional brain networks or connectomes were estimated and characterized by graph metrics to identify differences between the groups in global and regional connectivity, its evolution with age, and its influence on cognition. Munoz-Moreno et al. (2018) reported in detail on the early, 5 month time point and demonstrated important structural connectivity alterations and neurocognitive impairments in tg rats before the presence of significant concentration of Aβ plaques. At a global level, the structural connectome showed lower integration and segregation in tg than in control rats, pointing to a different pattern of anatomical connections in subjects developing AD. Decreased functional connectome properties were observed in amygdala, VTA and insular cortex, regions related to reward, memory, and sensory performance, known to be altered in patients with AD or mild cognitive impairment. In a follow-up study from the same authors (Munoz-Moreno et al., 2020), data from the entire longitudinal study (5, up to 18 months) were analyzed and showed that aging had a bigger impact on the structural connectome of the TgF344-AD than of the control animals. Moreover, changes in the structural network, already observed at early age, significantly influenced cognitive outcome of transgenic animals while FC showed more impact in the cognitive performance of control subjects. The training and the repetition of the cognitive task in the current study could lead to a learning effect, increasing the cognitive reserve of these animals and therefore preserving their functional connectivity.

### Dynamic Functional Connectivity Patterns: Brain State Changes in AD

Exploring time-varying connectivity networks in neurodegenerative disorders is a recent field of research in functional MRI (Sendi et al., 2021). RsfMRI in patients of Alzheimer's disease (AD) has identified DMN and TPN disruptions as promising biomarkers for AD (Mevel et al., 2011). Acquiring dynamic rsfMRI (drsfMRI) allows the detection of transient brain states such as the quasi-periodic patterns (QPPs) of neural activity describing recurring spatiotemporal patterns that display DMN with TPN in anti-correlation and could thus provide new insights into AD network dysfunction. An alternative drsfMRI analysis method is the resting-state co-activation patterns (CAPs) that represent instantaneous and transient brain configurations that are likely contributors to the emergence of commonly observed resting-state networks and QPPs. Both QPPs (Belloy et al., 2018) and CAPs (Adhikari et al., 2021) were extracted from the drsfMRI data from 18 months old tg2576 mice and significantly improved the genotypic classification compared to conventional FC measures obtained in the same cohort. These promising diagnostic candidate drsfMRI biomarkers for AD need further studies on early pre-plaque stages to see their value as early prognostic markers. van den Berg et al. (2021) reported in 4months old (pre-plaque stage) TgF344-AD rats significant spatial and temporal alterations in QPPs. In the WT littermates, the basal forebrain is co-activated with the cingulate cortex while this co-activation is greatly reduced in the TgF344-AD rats. The basal forebrain has been implicated as an important orchestrator of whole-brain network activity as one of the first regions affected by AD in humans. These results show that spatiotemporal changes in QPPs together with altered basal forebrain activity could be a potential signature to identify early onset changes at the network level.

### Network Changes in Huntington's Disease

Huntington's disease (HD) is a neurodegenerative disorder with an autosomal dominant inheritance caused by abnormal expansion of CAG repeats in the exon 1 of the huntingtin gene. Mutant htt (m-htt) is toxic to brain cells through combination of gain and loss of function mechanisms and leads to dysfunction and death of predominantly cortical and striatal neurons. A variety of tg animal models with different amount of CAG repeats and thus different timings of onset and severity of the disease have been developed (for review see Farshim and Bates, 2018).

As HD is a genetic disease, treatment of HD is likely to be most beneficial in the early, possibly pre-manifestation stage. RsfMRI may be the translational method of choice to develop biomarkers reflecting early neuronal dysfunction. However, only a few rsfMRI studies in animal models have been reported so far. Li et al. (2017) analyzed FC and its correlation to brain atrophy, as well as motor function in 5 months old N171-82Q HD mice. In the HD mice, the weaker intra-striatum connectivity was positively correlated with striatal atrophy, while striatum-retrosplenial cortex connectivity was negatively correlated with striatal atrophy. The regional FC had no significant correlation with motor deficits in HD mice, suggesting that altered brain connectivity detected by rsfMRI might provide indeed an early disease biomarker in HD. Using dynamic rsfMRI in the Q175 mouse model, Vasilkovska et al. (2021) analyzed the static FC but also the temporal fluctuations in FC by identifying QPPs. FC could only pick up genotypic difference at manifest stages while QPPs showed already a deviation from the wild-type mice at the pre-manifest stage (3 months) with a significant hyperconnectivity of striatal regions. This illustrates also in this disease model the higher sensitivity of dynamic rsfMRI as compared to static rsfMRI.

Other studies focused rather on restoring circuit function in HD. Fernandez-Garcia et al. (2020) used rsfMRI to map striatal network dysfunction in symptomatic 20 week old R6/1 mice in order to modulate the activity of a specific cortico-striatal circuit by optogenetically induced glutamate release from secondary motor cortex terminals in the dorsolateral striatum. Using MR spectroscopy to monitor glutamate release, electrophysiology, behavioral tests and IHC, they could prove amelioration of HD symptoms in symptomatic HD mice, raising hope for an effective therapeutic strategy. Chang et al. (2018) focused on another aspect of the disease, the hyperexcitability disrupting the structural and functional connectivity. Using different rsfMRI metrices in the YAC128 mouse model, they could demonstrate that a treatment with the NMDA antagonist memantine in the drinking water starting at 2 months of age until the day of the MRI scan at 10.5 months helps to reduce excitotoxicity and normalizes the FC in the YAC128 mouse model. This shows that rsfMRI is an excellent translational method to study pharmaceutical effects in brain circuitry.

## Network Changes After Stroke

Stroke is still today one of the major diseases with permanent disabilities and even death in the Western World (Katan and Luft, 2018). Today, treatments are limited to thrombolysis or thrombectomy during the acute phase and rehabilitation measures later. Particularly for the causally based rehabilitation approach, a better understanding of the impact of the focal lesion on the whole brain is needed. While early stroke studies focused on the directly affected tissue area, the ischemic territory, more recent investigations widened their view increasingly to include the whole brain in their analysis, monitoring the reciprocal influences between the primary lesion area and distant brain regions with neuronal network concepts.

### Structural Networks

**Figure 5:**
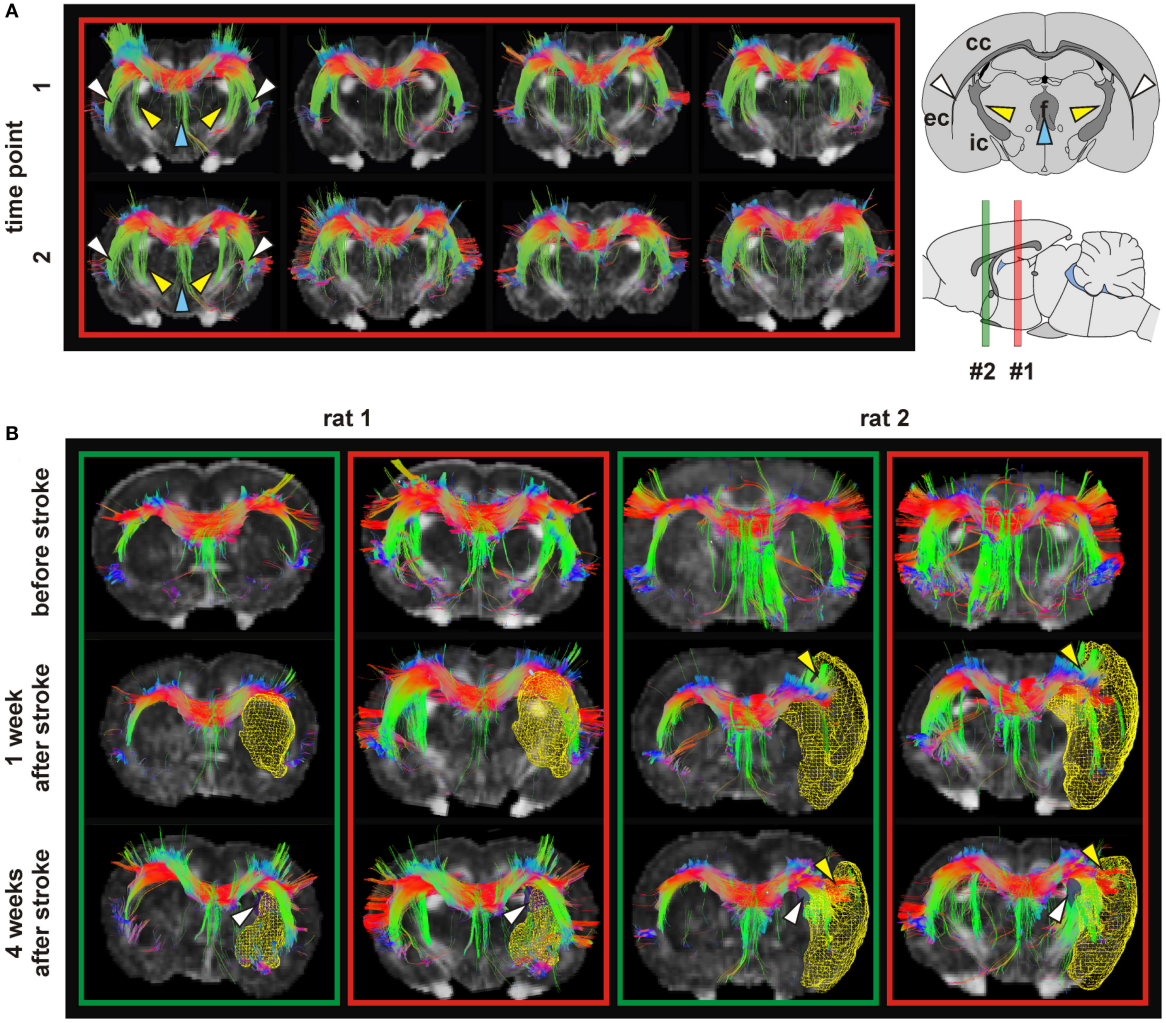
Tractography in healthy and ischemic conditions. **(A)** Tractography of four healthy rat brains (columns) with corpus callosum (CC) as seeding area at two time points (t1, t2) 4 weeks apart. In all cases, the fibers of the CC followed the white matter through the internal capsule (IC) (yellow arrows), external capsule (EC) (white arrows) and fornix (blue arrows). **(B)** Tractography in two rats before stroke, and 1 and 4 weeks after stroke. In the green boxes, the reference slice is close to bregma, corresponding to slice #2 on the sagittal scheme. For the red boxes the reference section is more caudal, indicated by position #1 on the scheme. Lesion volumes (determined from T2-weighted MRI) are depicted as yellow mesh. Rat 1 developed a stroke in the caudate putamen (CPu), and rat 2 in the CPu and cortex. At 4 weeks post stroke, a modification of the connection between CC and IC (white arrows) is seen. In rats with stroke in CPu and cortex (rat 2) an extension of the CC into the cortex separating the infarct area and the rest of the cortex (yellow arrows) was observed. Figures show color coded tracts in the horizontal (red), vertical (green) and transversal (blue) direction, indicated in the arrow schematic at top right. Reproduced with permission from Po et al. (2012).

Over the past decade, many clinical studies explored particularly the motor system (for an excellent review cf. Grefkes and Fink, 2014). While task-related or resting-state functional connectivities have found wide-spread attention, both in clinical and in animal experimental studies, the underlying structural networks and their changes after stroke are the focus of only a small number of rodent stroke studies. In a rat stroke model, Po et al. (2012) analyzed the signaling pathway through the thalamus to the sensorimotor cortex with DSI fiber tracking while monitoring the electrical forepaw stimulation in the primary somatosensory cortex with task-related fMRI. These authors reported an early reorganization of the ipsilesional internal capsule, followed by transcallosal new fiber connections, in parallel with recovery of the cortical activity to the forepaw stimulation (Figure 5). Interestingly, in rats without this transhemispheric new fiber bundle, no return of cortical activity was noted upon forepaw stimulation. Spontaneous generation of new transhemispheric fibers connecting the contralateral striatum to the ischemic territory as well as new fibers between perilesional tissue and thalamus have been reported by Granziera et al. (2007). Existence of these new DTI tractography-derived fibers was supported by staining of the plasticity-related protein GAP43 in the corresponding areas. Applying DSI to a mouse stroke model, Hafeneger (2013) described a complete loss of cortical fiber connections in the ipsilateral cortex 1 week after stroke. But after 4 weeks, new fiber connections from the cortex indicated spontaneous return of structural organization (cf. Fig. 22.2 in Aswendt et al., 2019). In a photothrombotic stroke mouse model, Aswendt et al. (2021b), using atlas-based whole-brain structural connectivity and *ex vivo* histology, revealed secondary neurodegeneration in the ipsilesional brain areas, mostly in the dorsal sensorimotor area of the thalamus. More importantly, these authors reported a lesion size-dependent increase in structural connectivity between ipsilesional cortex on the one hand and the contralesional primary motor cortex and thalamus, a new fiber connection developing during 4 weeks after stroke induction. The involvement of the contralesional hemisphere was associated with improved functional recovery relative to lesion size, in accordance with earlier findings by Murphy and Corbett (2009). In a filament occlusion stroke mouse model, including stem cell grafting for treatment, Green followed structural and functional network changes combining DSI and resting-state fMRI in a 12 week longitudinal study (Green et al., 2018). In their matrix-based analysis of the structural networks, these authors reported fiber density increases between cortex and white matter regions, predominantly on the ipsilateral hemisphere. Generation of these increased fiber connections were found independent of stem cell grafting, pointing to a spontaneous reaction of the brain to the primary ischemic event. In a very recent study, Pallast et al. (2020) applied atlas-based graph theoretical analysis to DTI fiber tracking data in a mouse model of photothrombotic stroke. These authors observed breakdown of connections between regions directly affected by the ischemic cortical lesion, but also detected substantially increased fiber strength between S1 somatosensory cortex and corticospinal tract (CST), starting at 2 weeks post stroke. This is supported by observations of substantial axonal sprouting in the CST after stroke in the mouse (Reitmeir et al., 2011). Moreover, this graph-based analysis approach detected long-range re-routing of intra- and interhemispheric connections related to improved sensorimotor behavior (Pallast et al., 2020).

### Functional Networks

While behavioral tests demonstrate functional deficits or recovery phases, assessments of functional brain networks contribute to the better understanding of the underlying mechanisms for the observed functional derangements. For this purpose, resting-state fMRI provides the ideal whole-brain functional neuronal connectome. In a rat stroke model, van Meer et al. (2010, 2012) reported a loss of interhemispheric connectivity of the primary sensorimotor regions. Acutely, a strong reduction of functional connectivity strength was observed, followed by a trend toward re-normalization. The authors described that spontaneous recovery of sensorimotor function was related to improvement of interhemispheric functional connectivity of the sensorimotor regions. Interestingly, this normalization of the interhemispheric connectivity was complete for small ischemic lesions, but it remained only partially normalized for large lesions.

**Figure 6:**
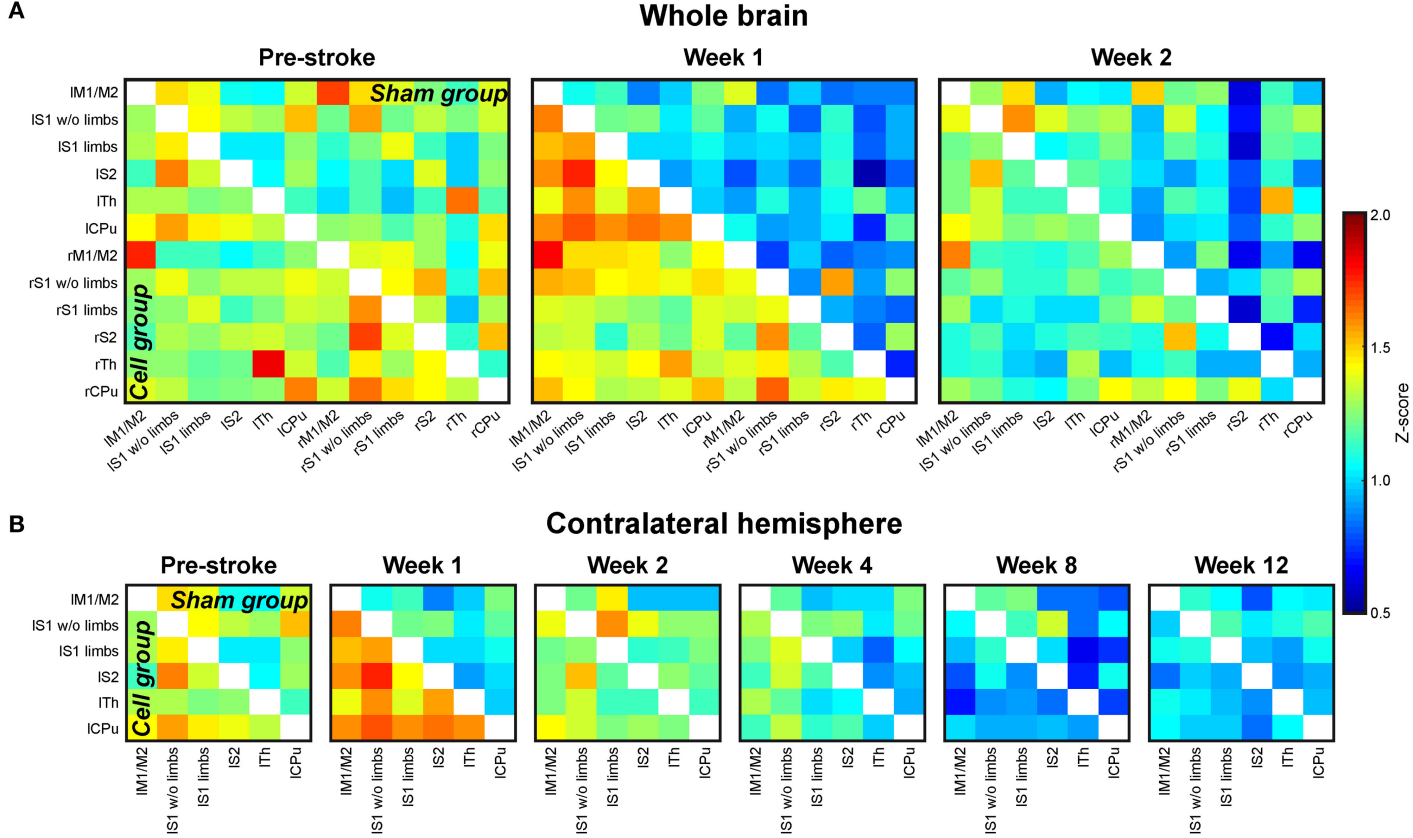
Cross-correlation z-score matrices of sensorimotor functional connectivity. **(A)** Z-score matrices of the whole mouse brain sensorimotor functional network before (pre-stroke) stroke induction and at 1 and 2 weeks after stroke induction. Directly following stroke, correlation strengths are decreased in the sham group (stroke without cell treatment; upper matrix triangle). The effect is strongest for the ipsilateral (right) hemisphere. In animals with stroke and stem cell implantation into the cortex adjacent to the ischemic lesion (cell group; lower matrix triangle), no reduction in the functional network is observed. **(B)** Z-score matrices of the sensorimotor networks on the contralateral hemisphere during the whole 12 weeks of observation. Similar to the ischemic hemisphere, but less pronounced, the sham group shows a connectivity decrease, continuously becoming more severe during the 12 weeks. In the stem cell group, the implantation stabilizes the connectivity strength still at week 2. Then, the functional connectivity continually decreases, approximating the lower value matrices of the sham group between week 8 and 12. “l” prefix, left hemisphere; “r” prefix, right hemisphere; M1/M2, primary/secondary motor cortex; S1w/o limbs, S1 somatosensory cortex without limb representation area; S1 limbs, limb representation area of S1; S2, secondary somatosensory cortex; Th, thalamus; CPu, caudate putamen. Reproduced with permission from Green et al. (2018).

In a mouse stroke model using the filament occlusion technique, Green et al. (2018, 2019a) monitored both functional and structural neuronal networks during 12 weeks following stroke induction. They found a sharp decrease of functional sensorimotor network strength which extended into the whole contralateral hemisphere, with this hypoconnectivity persisting for the whole 3 months observation period. In a separate group of animals, human neural stem cells were implanted into the vicinity of the ischemic cortex. Interestingly, this stem cell treatment resulted in a direct stabilization of the pre-stroke functional network strength (Figure 6). However, this stabilization was lost in animals with large lesions, extending into the graft location at chronic times and thus reducing the graft vitality. The direct stem cell induced stabilization of the functional network was associated with a paracrine effect of the graft while neuronal cell replacement could be excluded as a reason due to the necessary long period for differentiation into mature neurons (Tennstaedt et al., 2015). In a follow-up study, a beginning hypoconnectivity was noted in the late chronic phase despite preservation of graft vitality. From this it was concluded that quantitative graft vitality is a necessary but not sufficient criterion for persistent functional network stabilization (Green et al., 2019a). In their combination of functional with structural network assessment after stroke, the structural network changes were found independent of stem cell treatment. These results indicated that stem cells can influence, *via* paracrine effects, the functional networks, while observed structural changes are mainly stimulated by the ischemic event (Green et al., 2018).

Functional connectivity was also investigated in a mouse model of distal occlusion of the middle cerebral artery (Minassian et al., 2019) that resulted in small, circumscribed cortical ischemic territory (Minassian et al., 2020). In this stroke model of moderate severity, a hyperconnectivity of the sensorimotor networks of both hemispheres was observed, continuously further increasing during the 12 weeks observation period following stroke induction. Here again, as in the severe stroke model (Green et al., 2018, 2019a), grafting of human neural stem cells lead to a stabilization of the pre-stroke functional network strength, persistent over the whole observation period. The hyperconnectivity in the untreated stroke animals was interpreted as a hallmark of moderate stroke severity.

Combining mouse models of distal MCA occlusion, of photothrombotic stroke, and clinical data of patients with moderate stroke severity, Blaschke et al. (2021) analyzed functional connectivity data with graph theoretical approach. They reported hyperconnectivity with closely similar features for all strokes of mild to moderate severity in both species, with graph theory indicating decrease of global communication structures within the whole brain. This first true translational stroke study further strengthens the full translational relevance of animal experimental studies investigating functional network disturbances after cerebral disease.

### Interventional Modulation of Functional Network Changes

In addition to the above already discussed stem treatment of stroke by Green et al. (2018, 2019a) and by Minassian et al. (2020) further studies have recently appeared dealing with modulation of functional network changes after stroke or in investigations into the cellular roles during the pathophysiological cascade of events. A multi-center consortium using metabolic PET imaging and resting-state functional MRI investigated the influence of certain diet compositions on the outcome after stroke (Wiesmann et al., 2017). They reported increased functional connectivity strength between different brain regions, in particular the interhemispheric motor cortices, upon addition of a multicomponent diet.

Investigating the influence of the gut-brain-axis on the functional neuronal networks, Aswendt et al. (2021a) used mice, raised completely germ-free, and normally colonized mice under naïve conditions and after photothrombotic cortical stroke. They observed a strong, brain-wide hyperconnectivity in germ-free animals. Graph theoretical analysis revealed significantly higher values in germ-free animals, indicating a stronger and denser global network but with less structural organization. Breakdown of network function after stroke equally affected germ-free and normally colonized mice. This first study points to a substantial impact of bacterial colonization on brain-wide function.

The role of reactive astrogliosis in functional connectivity after photothrombotic stroke was studied by comparing wild-type with GFAP^−/−^Vim^−/−^ transgenic mice presenting attenuated reactive gliosis (Aswendt et al., 2021c). Four weeks after stroke, these transgenic mice showed impaired recovery of sensorimotor function and aberrant restoration of global neuronal connectivity. They also exhibited maladaptive plasticity responses, demonstrated by higher number of lost and newly formed functional connections between primary and secondary targets of cortical stroke regions, and further supported by increased peri-infarct expression of the axonal plasticity marker GAP43.

## Added Value by Combining Functional and Structural Network Readouts

It is the general belief that the structural connectome acts as the platform on which the functional connectome works. Several studies on human structural and functional networks proposed that functional networks indirectly reflect the architecture of the structural connectome (Honey et al., 2009; van den Heuvel et al., 2009; Zhang et al., 2010). However, there is very little systematic investigation whether this belief is not slightly simplified and, if generally correct, under what conditions it will hold. This is of particular interest for the study of disease- or injury-induced disturbances of brain networks. Two recent publications have approached this issue of the structural basis of the functional connectome (Straathof et al., 2019, 2020). In their review, Straathof et al. (2019) discussed the quantitative relationship between structural and functional network strengths in mammalian brain. This study, based predominantly on human data of variable experimental condition and spatial resolution, concludes that structural connectivity provides the architecture for functional networks. However, the authors warrant that “methodological differences between the included studies complicate the comparison across studies, which emphasizes the need for validation and standardization in brain structure-function studies” (Straathof et al., 2019). In a follow-up study rsfMRI-derived functional networks of rat brain were compared with structural networks derived from DTI tractography or neuronal tracers. In this investigation, Straathof et al. (2020) found a strong correlation with DTI based tractography for sensorimotor and default mode networks, but only weak correlation for heterotopic interhemispheric and long-range intrahemispheric structural connectivity. Grandjean et al. (2017b) investigated the structural basis of the functional connectome of the mouse. For this purpose, they compared the functional network data derived from resting-state fMRI to the structural network data provided by the Allen Brain Connectivity Atlas (Oh et al., 2014), which was compiled from monosynaptic viral tracer experiments. According to Grandjean's analysis, functional connectivity between interhemispheric homotopic cortical and hippocampal areas as well as the cortico-striatal pathways are reflected in monosynaptic connections. However, for certain subcortical networks, the functional connectivity requires polysynaptic pathways, not sharing direct anatomical connections.

Karatas et al. (2021) recently reported comparison of structural and functional connectivity in two healthy mouse strains (C57BL/6N and BALB/cJ) using DTI and rsfMRI. They observed inter-strain differences regarding fiber density within frontal cortices, along cortico-striatal, thalamic and midbrain pathways. Interestingly, these authors found that for homotopic cortical areas, differences in structural connection strength between the strains were not followed by corresponding functional connectivity strength, pointing toward the possibility of decoupling patterns between structural and functional networks in some areas.

There are three reports on various Alzheimer's disease models investigating both structural and functional network changes. In the tau model used by Green et al. only marginal structural changes were observed during tau regulation while massive functional hypoconnectivity across the whole brain was described (Green et al., 2019b). In two studies by Munoz-Moreno et al. on a rat AD model, the authors analyzed all datasets only with global graph theoretical metrics, thus not describing comparison of individual connections. They reported lower integration and segregation of the structural networks, pointing to different patterns of anatomical connections. The structural differences did, however, not lead to changes in functional metrics (Munoz-Moreno et al., 2018, 2020).

In the only rodent stroke study including structural and functional connectivity data by DSI and resting-state MRI, Green et al. (2018) reported appearance of functional network disturbances directly following stroke induction while structural changes were detected only with delay of some weeks. Moreover, in this study, functional connectivity was modulated by stem cell treatment after stroke while structural changes of fiber loss or increases were proposed to be exclusively a response of the brain to the primary lesion, independent of treatment inclusion. In a recent review, Dijkhuizen et al. (2014) emphasized again that the relationship between structural and functional network changes “remains incompletely understood.”

In conclusion, the above presented reports warrant that for a complex understanding of the brain's responses to disease the combined monitoring of changes of the structural and functional connectome are strongly advisable.

## Discussion/Summary

We have discussed changes of structural and functional neuronal networks in disease models for investigations into neurodegeneration, in particular Alzheimer's disease and Huntington's disease, and into stroke, and found them well-suited for detection of early biomarkers and markers for disease progression. These preclinical studies can help to unravel the pathological underpinning of aberrant rsfMRI and DTI patterns.

### Neurodegeneration and Stroke

In Alzheimer's disease models, we found only marginal information on structural network changes over time. However, there are many DTI studies in AD and even in HD models that focused rather on DTI parameter changes as a readout for pathology or that were based on *ex vivo* data and were therefore not included in this review. Early phases of the disease are characterized by functional hyperconnectivity while late phases at severe progression of the disease present profound hypoconnectivity. Interestingly, results on altered functional networks point toward a toxic effect of soluble Aβ and of soluble tau already. Thus, hyperconnectivity was reported to occur at phases of only soluble Aβ indicating its toxic effect already before plaque formation (Shah et al., 2016c; Latif-Hernandez et al., 2019) and in prodromal stages of tauopathy (Degiorgis et al., 2020), and hypoconnectivity was observed equally in models with soluble and with tangled human tau (Green et al., 2019b). These observations may become possible early, clinically relevant diagnostic markers, independent of further pathological indications.

In stroke investigations, generation of new (trans-)hemispheric fiber connections to the ischemic territory was observed by several authors, pointing toward strong plasticity and reorganization response of the brain to the primary lesion. Similar to the distinction of early and late disease progression phases in Alzheimer's disease by functional network changes, in stroke mild/moderate and severe cases are distinguished by increase and decrease, respectively, of functional networks.

The presentation of early AD phase by hyperconnectivity, late phase by hypoconnectivity and the specific alterations in the DMN reflects well the clinical findings (Mevel et al., 2011; Quiroz et al., 2015; Badhwar et al., 2017); also excellent agreement of mild stroke in mice and men was reported (Blaschke et al., 2021). These findings emphasize the clinical relevance of the animal experimental investigations and support the translational robustness of the applied disease model systems.

### Interpretation of Functional Connectivity Alterations

In structural networks, often existence or loss of fiber connections between two areas are of interest. When the terms hypo- and hyper-connectivity are used in relation to structural network analysis, they refer to increased or decreased fiber densities.

**Figure 7:**
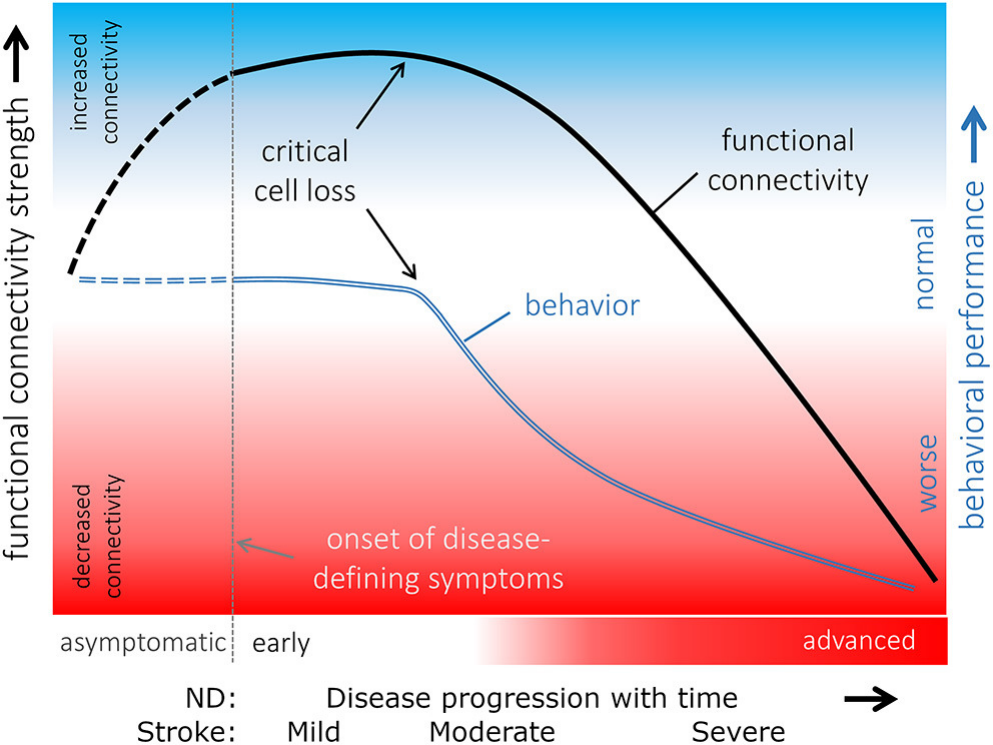
Hypothetical model of functional connectivity alterations in association with behavioral performance in the course of neurodegeneration. The pattern of functional connectivity changes (black line) and its association with behavior (blue line) indicated that functional connectivity increases in a potentially compensatory response to ongoing cell degeneration in order to maintain “normal” behavioral performance as long as possible. When a critical cell loss is reached, i.e., the functional reserves are exhausted, behavioral performance declines, and functional connectivity decreases upon a disconnection syndrome with poor behavioral performances presented in an advanced disease state. The connectivity curve follows the progression in neurodegenerative diseases (ND). For stroke, it reflects the different severity stages from mild to severe. It remains open whether functional connectivity is already altered in an asymptomatic phase of an underlying neurodegenerative pathological process (dashed lines). Adapted with permission from Gorges et al. (2017).

In relation to functional networks, hyperconnectivity has been described as a hallmark of neurological disturbance (Hillary et al., 2014). Indeed, such hyperconnectivity has been reported in clinical studies by various authors (Hillary et al., 2014; Iraji et al., 2015; Liu et al., 2018b). Recently, a hypothetical model of connecting disease progression with functional network alterations was proposed by two labs (Gorges et al., 2017; Hillary and Grafman, 2017). Based on this model, functional connectivity increase (i.e., hyperconnectivity) acts as “a potentially compensatory response to ongoing cell degeneration in order to maintain normal behavioral performance” (Figure 7) (Gorges et al., 2017). This hyperconnectivity, also seen in cognitive studies of Alzheimer's patients (Gregory et al., 2017; Cabeza et al., 2018; Behfar et al., 2020), is often interpreted as a compensation (or reserve capacity) status of the brain in response to cerebral disease (Fornito et al., 2015; Hillary and Grafman, 2017). As the disease progresses and its severity or damage increases, the system reaches a level where the functional network collapses, reflected by hypoconnectivity. While in neurodegenerative diseases the connectivity strength changes from hyper- to hypoconnectivity with disease progression, in stroke the same change appears to follow with different severity levels from mild—moderate—severe. It must be cautioned, however, that the patterns of hyper- and hypoconnectivity, respectively, do not provide the complete picture of the disease progression. Additional analytical approaches by graph theory on several metrics such as whole-brain modularity and clustering coefficients may help to further elucidate different brain states of adaptive or maladaptive condition, thereby further differentiating in particular the hyperconnectivity phase of the disease. It may also allow to better differentiate brain-lesion responsive hyperconnectivity states for their effectiveness of disease compensation or reserve capacities (Fornito et al., 2015). These authors also discuss the potential of using data on structural and functional network changes for the generation of predictive models of the spread and functional consequences of brain disease.

Interestingly, a few experimental approaches including therapeutic treatment (cf. above both after stroke and after AD) reported rapid normalization of the functional connectivity strength. The underlying mechanisms for these observations and their interpretation within such a hypothetical model still need future investigations.

Finally, it is of note that systematic combinations of both structural and functional network assessments during disease progression are still sparse. But future attention in this field is expected in order to generate new understanding on how the brain responds to aging, cerebral diseases or injury, potentially distinguishing structural and functional brain plasticity.

## Author Contributions

All authors listed have made a substantial, direct, and intellectual contribution to the work and approved it for publication.

## Funding

AVdL gratefully acknowledged support by the Hercules Foundation funding (Belgium, Grant Agreement AUHA/012) and Flemish Impulse funding (Grant Number 42/FA010100/1230).

## Conflict of Interest

The authors declare that the research was conducted in the absence of any commercial or financial relationships that could be construed as a potential conflict of interest.

## Publisher's Note

All claims expressed in this article are solely those of the authors and do not necessarily represent those of their affiliated organizations, or those of the publisher, the editors and the reviewers. Any product that may be evaluated in this article, or claim that may be made by its manufacturer, is not guaranteed or endorsed by the publisher.
